# 

**Published:** 1878-10-20

**Authors:** 


					﻿fa
03
T*
fa
fa
W
03
M
J
O
, fa
Q
5
6
O
Jx
O
fa
<!
03
fa
O
fa
co
I—I
B
fa,
fa
O
GO
S
fa
<!
fa
o
fa
fa;
fa
fa
fa
fa
'0
fa
03
fa
fa
co
<<
fa
fa
fa
VOL. VIII. ?-. < - OCT. 20, 1878. .	No. 10.
THE SOUTHERN
MEDICAL RECORD.
JAonthly jJout\nal of Practical Medicine.
Terms : 82 OO per annum In advance. Club Rates, 5 copies
88.00. Single copies 20 cents.
“Qtc/cgtw prjecipies esto brevis.”
1
00
fa
O
fa
fa
Q
O
fa
fa
i—i
Q
f>
fa
i—i
O
fa
GO
fa
fa
a
H
i—<
e
f
i—i
h
w
GO
GO
o
fa
I—(
Q
i—<
fa
W
fa
_______ ESTABLISHED IN 187O._____________________
ATLANTA. GA.:
Sunny South Steam jTOblkhing House.
Address R. C. WORD, M. D., Managing Editor, Atlanta, Ga.
				

